# Accelerated dual-venc 4D flow MRI with variable high-venc spatial resolution for neurovascular applications

**DOI:** 10.1002/mrm.29306

**Published:** 2022-06-26

**Authors:** Maria Aristova, Jianing Pang, Yue Ma, Liliana Ma, Haben Berhane, Vitaliy Rayz, Michael Markl, Susanne Schnell

**Affiliations:** 1 Department of Radiology, Northwestern University Feinberg School of Medicine, Chicago, Illinois, USA; 2 MR R&D and Collaborations, Siemens Medical Solutions USA Inc., Chicago, IL, USA; 3 Department of Radiology, Shengjing Hospital of China Medical University, Shenyang, China; 4 Department of Biomedical Engineering, Northwestern University McCormick School of Engineering, Evanston, Illinois, USA; 5 Weldon School of Biomedical Engineering, Purdue University College of Engineering, West Lafayette, Indiana, USA; 6 Institut für Physik, Universität Greifswald, Greifswald, Germany

**Keywords:** 4D flow, acceleration, acquisition, dual-venc, neurovascular

## Abstract

**Purpose:**

Dual-velocity encoded (dual-venc or DV) 4D flow MRI achieves wide velocity dynamic range and velocity-to-noise ratio (VNR), enabling accurate neurovascular flow characterization. To reduce scan time, we present interleaved dual-venc 4D Flow with independently prescribed, prospectively undersampled spatial resolution of the high-venc (HV) acquisition: Variable Spatial Resolution Dual Venc (VSRDV).

**Methods:**

A prototype VSRDV sequence was developed based on a Cartesian acquisition with eight-point phase encoding, combining PEAK-GRAPPA acceleration with zero-filling in phase and partition directions for HV. The VSRDV approach was optimized by varying *z*, the zero-filling fraction of HV relative to low-venc, between 0%–80% in vitro (realistic neurovascular model with pulsatile flow) and in vivo (*n* = 10 volunteers). Antialiasing precision, mean and peak velocity quantification accuracy, and test–retest reproducibility were assessed relative to reference images with equal-resolution HV and low venc (*z* = 0%).

**Results:**

In vitro results for all *z* demonstrated an antialiasing true positive rate at least 95% for *R*_PEAK−GRAPPA_ = 2 and 5, with no linear relationship to *z* (*p* = 0.62 and 0.13, respectively). Bland–Altman analysis for *z* = 20%, 40%, 60%, or 80% versus *z* = 0% in vitro and in vivo demonstrated no bias *>*1% of venc in mean or peak velocity values at any *R*_ZF_. In vitro mean and peak velocity, and in vivo peak velocity, had limits of agreement within 15%.

**Conclusion:**

VSRDV allows up to 34.8% scan time reduction compared to PEAK-GRAPPA accelerated DV 4D Flow MRI, enabling large spatial coverage and dynamic range while maintaining VNR and velocity measurement accuracy.

## 1 | INTRODUCTION

4D flow MRI has been used to characterize the hemodynamics of neurovascular arterial and venous processes including aneurysms,^1–4^ atherosclerotic disease,^5–7^ pulsatile tinnitus,^8^ intracranial arteriovenous malformations,^9–12^ and fistulae^13^ among others.^14–16^ However, neurovascular vessels are anatomically variable and small relative to the total intracranial volume, and velocities may vary by an order of magnitude between arteries and veins.^12^ Thus, single-velocity encoded 4D flow does not accurately capture the full range of intracranial blood flow velocities, and conventional anti-aliasing is difficult due to small numbers of voxels within vessel cross-sections.

Dual-velocity encoded (dual-venc or DV) 4D flow MRI addresses this by acquiring interleaved low-venc (LV) and high-venc (HV) images,^17^ achieving wide velocity dynamic range while maintaining a high velocity-to-noise ratio (VNR).^18^ DV is well-suited for 4D flow measurement in neurovascular applications.^6,19–21^

However, the challenge of balancing spatiotemporal resolution remains. Previous work has shown that isotropic spatial resolution of (0.8–1.0 mm)^3^ is needed for accurate quantification of intracranial flow^22^ and for advanced quantification methods including pressure estimation.^23^ The ability to reduce scan time by compromising temporal resolution is further limited under k-t acceleration approaches such as PEAK-GRAPPA^24^ and by the already decreased maximum temporal resolution of DV. Rather than reducing DV spatiotemporal resolution, an alternative method of time savings is to reduce resolution in the HV acquisition only, while preserving temporal and spatial resolution in the LV data.

This principle was applied in a five-point balanced flow encoding scheme, where the fifth flow encoding, used to correct velocity aliasing, was acquired at reduced spatial and temporal resolution and enabled a factor of 1.6 VNR gain with few aliasing artifacts.^25^ This approach was systematically investigated using radial PC-VIPR, which achieved a factor of 2.88 VNR gain with separately acquired, retrospectively undersampled HV used to correct LV aliasing.^26^ As predecessors to the current work, HV undersampling combined with elliptical k-space sampling has been previously implemented without advanced acceleration in 2D^27^ and 3D^28^ phase-contrast sequences with interleaved eight-point DV encoding, with HV undersampling accomplished by zero-filling in the phase encoding direction.

By contrast, the goal of this study was to develop and test an interleaved, three-directional DV sequence with independently prescribed LV and HV spatial resolution that preserves the dynamic range and VNR advantages of dual venc. The resulting Variable Spatial Resolution Dual Venc (VSRDV) sequence is an interleaved eight-point DV 4D Flow MRI sequence with integrated PEAK-GRAPPA acceleration and independently prescribed, prospectively undersampled spatial resolution of the HV acquisition. The hypotheses of this study are that DV scans with low-resolution HV data (with up to 80% zero-filling of HV k-space) will enable phase unwrapping of at least 95% of aliased voxels compared to a reference acquisition where the HV and LV spatial resolution is identical; and that such acquisitions will result in mean and peak velocity measurements within 15% of the corresponding values from reference acquisitions without undersampling (based on previously reported reproducibility of DV).^22^ These hypotheses were investigated in complex neurovascular geometries in vitro and in vivo.

## 2 | METHODS

### 2.1 | Pulse sequence and dual-venc velocity encoding

The prototype VSRDV sequence is based on an eight-point dual-venc 4D flow MRI acquisition scheme with Cartesian sampling, where four 3D k-spaces (phase reference and phase encoding in three orthogonal directions) are acquired in each cardiac timeframe for both LV and HV images, and a cardiac timeframe (8×TR) defines the temporal resolution. An eight-point encoding scheme was chosen to maintain constant temporal resolution throughout the acquisition, using a previously published TE-minimizing strategy.^29^

**FIGURE 1:**
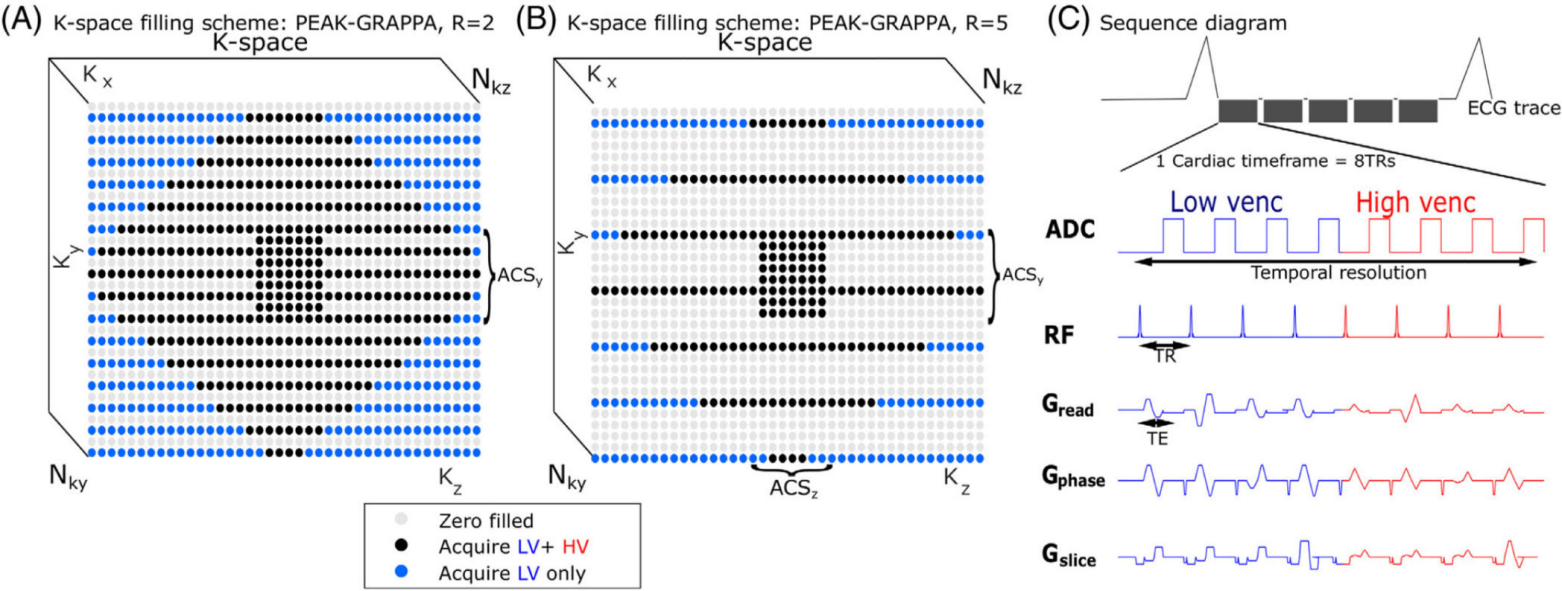
Schematic K-space acquisition scheme for single timeframe in VSRDV 4D Flow MRI with *z* = 40% and *R*_PEAK_-_GRAPPA_ = 2 acceleration (A) and *R*_PEAK_-_GRAPPA_ = 5 acceleration (B), with points of k-space indicated by black dots having LV and HV acquired, those with light gray dots having both LV and HV omitted in PEAK-GRAPPA scheme, and blue dots denoting k-space lines having LV acquired and HV zero-filled. Zero-filled *K*_*y*_ lines are alternated throughout the temporal domain (not shown). C, Sequence diagram showing interleaved eight-point acquisition of LV and HV as in typical DV imaging

The sequence incorporates acceleration using PEAK-GRAPPA, an extension of GRAPPA parallel imaging to the time domain^24,30^ applied to both LV and HV. Undersampling due to PEAK-GRAPPA with *R*_PEAK−GRAPPA_ = 2 and 5 is shown schematically by light gray points in Figure 1A,B, respectively. In each case, k-space outside the central autocalibration signal (ACS) region is undersampled in a “cut-corner” elliptical k-space sampling approach.^31^ This approach has been extensively described for both an idealized phantom and for Cartesian gradient echo sampling,^32^ and previously implemented in combination with GRAPPA acceleration for single-venc 4D Flow MRI in the context of neurovascular applications.^5^ This sampling approach preferentially zero-fills k-space positions with high-frequency information, which is most likely to be redundant with the LV acquisition.

The user-defined PEAK-GRAPPA acceleration factor *R* describes the ratio of omitted to acquired lines outside the ACS region, so the true acceleration factor in each case is computed from the number of $k_{y}-k_{z}$ lines sampled in both the LV and HV k-spaces. For matrix dimensions $N_{ky}$ and $N_{kz}$ in the $k_{y}$ and $k_{z}$ directions respectively, the number of $k_{y}-k_{z}$ lines for a fully sampled k-space, ${\text{Lines}}_{\text{LV},\text{fully sampled}}$ is given by :

(1) $${\text{Lines}}_{\text{LV},\text{fully sampled}}=N_{ky}N_{kz}$$

For PEAK-GRAPPA-accelerated acquisition, with ACS_*y*_ and ACS_*z*_, the ACS lines in the $k_{y}$ and $k_{z}$ directions respectively, the number of $k_{y}-k_{z}$ lines is ${\text{Lines}}_{\text{LV,PEAK-GRAPPA}}$, denoting the number of LV lines acquired under typical PEAK-GRAPPA:

(2) $${\text{Lines}}_{\text{LV,PEAK-GRAPPA}}=\left(N_{ky}-{\text{ACS}}_{y}\right)\left(N_{kz}-{\text{ACS}}_{z}\right)\;(1/R)+{\text{ACS}}_{y}{\text{ACS}}_{z}$$

For fully-sampled LV and HV, the number of *k*_*y*_-*k*_*z*_ lines sampled in HV (Lines_HV_) is the same as Lines_LV_ and the resulting acceleration factor due to PEAK-GRAPPA, denoted by $R_{\text{PEAK-GRAPPA}}$ is:

(3) $$\begin{array}{l} R_{\text{PEAK-GRAPPA}}=\frac{{\text{Lines}}_{\text{LV},\text{fully sampled}}}{{\text{Lines}}_{\text{LV},\text{PEAK-GRAPPA}}}\\ \;=\frac{N_{ky}N_{kz}R}{\left(N_{ky}-{\text{ACS}}_{y}\right)\left(N_{kz}-{\text{ACS}}_{z}\right)+{\text{ACS}}_{y}{\text{ACS}}_{z}R}. \end{array}$$

In the VSRDV sequence, variable spatial resolution of the HV acquisition was implemented by additional zero-filling at the outer corners of k-space (blue points in Figure 1A,B). As per the sequence diagram (Figure 1C), at the center of k-space a reference and three orthogonal phase encodings are collected for both LV and HV within one cardiac time frame. At the peripheral corners of k-space, two different LV k-space lines (similar to segmented k-space sampling) are acquired per flow encoding direction and cardiac time frame while the corresponding HV lines are zero-filled. This reduces the total number of heartbeats required to fill k-space but preserves the temporal resolution of the reference with equal LV and HV resolution.

Therefore, all acquisitions are characterized by the combination of matrix size, ACS line numbers, $R_{\text{PEAK-GRAPPA}}$ (applied to both HV and LV) as above, and fraction of HV k-space that is zero-filled (*z*). The acceleration factor due to zero-filling is considered relative to the underlying sampling pattern. The acceleration factor due to zero-filling $R_{\text{ZF}}$ is defined as:

(4) $$R_{\text{ZF}}=\frac{2{\text{Lines}}_{\text{LV}}}{{\text{Lines}}_{\text{LV}}+{\text{Lines}}_{\text{HV}}}.$$

Then the total acceleration for PEAK-GRAPPA with zero-filling is

(5) $$R_{\text{total}}=\frac{{\text{Lines}}_{\text{LV},\text{fully sampled}}+{\text{Lines}}_{\text{HV},\text{fully sampled}}}{{\text{Lines}}_{\text{LV}}+{\text{Lines}}_{\text{HV}}}\;=\frac{2{\text{Lines}}_{\text{LV},\text{fully sampled}}}{{\text{Lines}}_{\text{LV},\text{PEAK-GRAPPAςZF}}+{\text{Lines}}_{\text{HV},\text{PEAK-GRAPPAςZF}}}\;=R_{\text{GRAPPA}}R_{\text{ZF}}$$

with ${\text{Lines}}_{\text{LV,PEAK-GRAPPA\&ZF}}$ and ${\text{Lines}}_{\text{HV,PEAK-GRAPPA\&ZF}}$ being the number of $k_{y}-k_{z}$ lines sampled in LV and HV, respectively, under the combination of PEAK-GRAPPA acceleration and HV zero-filling.

In the simplest case, zero-filled lines do not overlap with ACS lines. In that case, the total number of k-space segments acquired along the *k*_*x*_ and *k*_*t*_ directions in the HV is:

(6) $${\text{Lines}}_{\text{HV}}={\text{Lines}}_{\text{LV}}-zN_{ky}N_{kz}(1/R)$$

so

(7) $$R_{\text{ZF}}=\frac{2{\text{Lines}}_{\text{LV}}}{2{\text{Lines}}_{\text{LV}}-zN_{ky}N_{kz}(1/R)}.$$

Then

(8) $$\begin{array}{c} R_{\text{total}}=R_{\text{ZF}}R_{\text{PEAK-GRAPPA}}\\ =\frac{2{\text{Lines}}_{\text{LV,PEAK-GRAPPA}}}{2{\text{Lines}}_{\text{LV,PEAK-GRAPPA}}-zN_{ky}N_{kz}(1/R)}\\ \left(\frac{{\text{Lines}}_{\text{LV,fully sampled}}}{{\text{Lines}}_{\text{LV},\text{PEAK-GRAPPA}}}\right)\\ =\frac{{2\text{Lines}}_{\text{LV,fully sampled}}}{2\text{Line}s_{\text{LV},\text{PEAK-GRAPPA}}-zN_{ky}N_{kz}(1/R)}\\ =\frac{2N_{ky}N_{kz}}{2\text{Line}s_{\text{LV},\text{PEAK-GRAPPA}}-zN_{ky}N_{kz}(1/R)}. \end{array}$$

For PEAK-GRAPPA-accelerated LV,

(9) $${\text{Lines}}_{\text{LV,PEAK-GRAPPA}}=\left(N_{ky}-{\text{ACS}}_{y}\right)\left(N_{kz}-{\text{ACS}}_{z}\right)(1/R)\;+{\text{ACS}}_{y}{\text{ACS}}_{z}$$

(10) Rtotal =RZF RPEAK-GRAPPA =2NkyNkz(Nky−ACSy)(Nkz−ACSz)(1R)+ACSyACSz−zNkyNkz(1R)=2RNkyNkz(Nky−ACSy)(Nkz−ACSz)+ACSyACSzR−zNkyNkz⋅

### 2.2 | In vitro experimental setup

**FIGURE 2:**
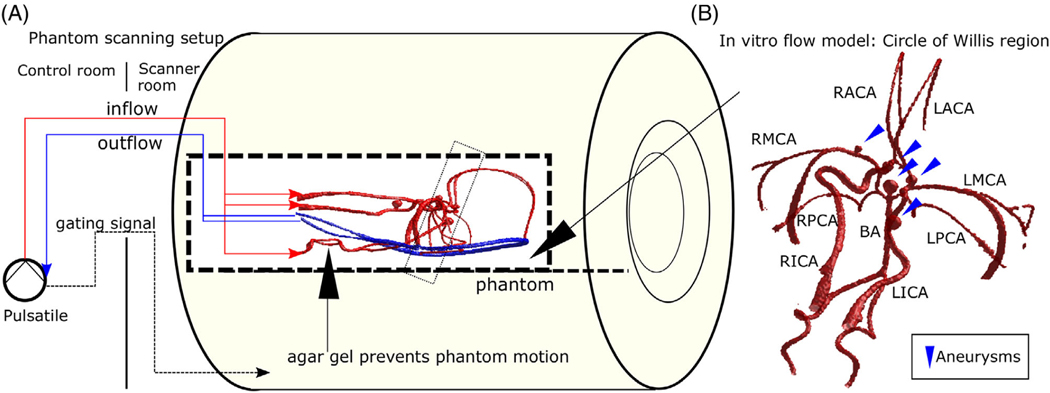
In vitro setup for neurovascular anatomical phantom with pulsatile flow (A), and detailed view of Circle of Willis region of the phantom (B)

Variable Spatial Resolution Dual Venc 4D Flow MRI was tested in vitro in a realistic neurovascular anatomical replica phantom with pulsatile flow (Figure 2A,B), consisting of a silicone model of the Circle of Willis with multiple aneurysms (COW01V03: United Biologics, CA, USA) integrated into a flow circuit with a pulsatile blood pump (model 1421, Harvard Apparatus: Holliston, MA, USA). A branched arrangement of inlet tubing supplied the left and right internal carotid arteries (ICAs) and vertebral arteries (VAs) of the phantom; branched outlet tubing collected outflow from the left and right sides of the phantom. Vessels include basilar (BA); right and left internal carotid (RICA, LICA); right and left middle cerebral (RMCA, LMCA); right and left anterior cerebral (RACA, LACA); right and left posterior cerebral (RPCA, LPCA); right and left proximal posterior cerebral (RPPCA, LPPCA); right and left posterior communicating (RPCOM, LPCOM) arteries. The silicone model was suspended in 0.5% agar gel to prevent motion, maintain a realistic spatial orientation, and provide static tissue for phase offset correction. Gadobutrol (Gadavist: Bayer AG, Germany) was added to 0.2% by volume in circulating water (*T*_1_ = 100 ms, *T*_2_ = 70 ms) and 0.1% in agar gel (*T*_1_ = 200 ms, *T*_2_ = 141 ms)^33^ to increase signal magnitude. The flow circuit was operated with approximately 1170 ms pulse interval and stroke volume 20 ml.

### 2.3 | In vivo cohort

Ten healthy volunteers (age 30±4 y, four female) were recruited in a prospective, institutional review board-approved study. Of these, eight subjects (age 30±4 y, three female) were scanned in two sessions each, 6–46 days apart, and two subjects were scanned in one session. Blood pressure was measured at each scan session to ensure the validity of test–retest reproducibility analysis.

### 2.4 | In vitro image acquisition

In vitro imaging was performed at 1.5T (MAGNETOM Aera, Siemens Healthcare, Erlangen, Germany) with TE = 3.7 ms, TR = 6.7 ms, flip angle 15^°^, venc_low_ = 55cm/s, venc_high_ = 110cm/s in all directions, and LV spatial resolution (0.8×0.8×0.8) mm^3^, $N_{ky}=160$, $N_{kz}=30$, ${\text{ACS}}_{y}=40$, and ${\text{ACS}}_{z}=8$. For each of *R*_PEAK−GRAPPA_ = 2 and 5, an acquisition with *z* = 0 (HV resolution equal to LV) was used as a reference for aliasing identification and velocity quantification. Other tested values of *z*, as well as the resulting *R*_total_ and time savings are tabulated in Table 1 according to Equation (10) above.

### 2.5 | In vivo image acquisition

In vivo imaging was performed at 3T (MAGNETOM Prisma, Siemens Healthcare, Erlangen, Germany) with flip angle 15^°^, ${\text{venc}}_{\text{low}}=55\text{cm}/\text{s}$, ${\text{venc}}_{\text{high}}=110\text{cm}/\text{s}$ in all directions, and $R_{\text{PEAK-GRAPPA}}=5$ to keep all acquisitions within 15minutes. LV spatial resolution was set to (1.0×1.0×1.0) mm^3^ (TE = 3.8 ms, TR = 6.5 ms) and decreased to (1.1×1.1×1.1) mm^3^ (TE = 3.7 ms, TR = 6.3 ms) if needed to accommodate subject head size in the phase encoding (left–right) direction with a consistent matrix size. Each scan session included acquisitions with *z* = 0%, *z* = 40% and *z* = 80% in that order, and with all other parameters, including number of cardiac time points, held constant.

### 2.6 | Dual-venc reconstruction

All DV images are reconstructed from a LV and HV image according to a previously published algorithm^17^ with minor modifications to the aliasing detection thresholds based on observed performance. For LV and HV images, we initially set the DV image equal to LV, then compute a difference image diff = HV−LV. The following substitutions are used to unwrap aliased voxels with up to 4 phases, using the venc value venc_low_ and assuming ${\text{venc}}_{\text{high}}=2*{\text{venc}}_{\text{low}}$:

$$\{\begin{array}{l} \text{DV}\left({\text{venc}}_{\text{low}}*1.0<\text{diff}<{\text{venc}}_{\text{low}}*2.8\right)=\text{LV}+2*{\text{venc}}_{\text{low}}\\ \text{DV}\left(-{\text{venc}}_{\text{low}}*2.8<\text{diff}<-{\text{venc}}_{\text{low}}*1.0\right)=\text{LV}-2*{\text{venc}}_{\text{low}}\\ \text{DV}\left({\text{venc}}_{\text{low}}*3.2<\text{diff}<{\text{venc}}_{\text{low}}*4.8\right)=\text{LV}+4*{\text{venc}}_{\text{low}}\\ \text{DV}\left(-{\text{venc}}_{\text{low}}*4.8<\text{diff}<-{\text{venc}}_{\text{low}}*3.2\right)=\text{LV}-4*{\text{venc}}_{\text{low}} \end{array}.$$

### 2.7 | In vitro image generation

**FIGURE 3:**
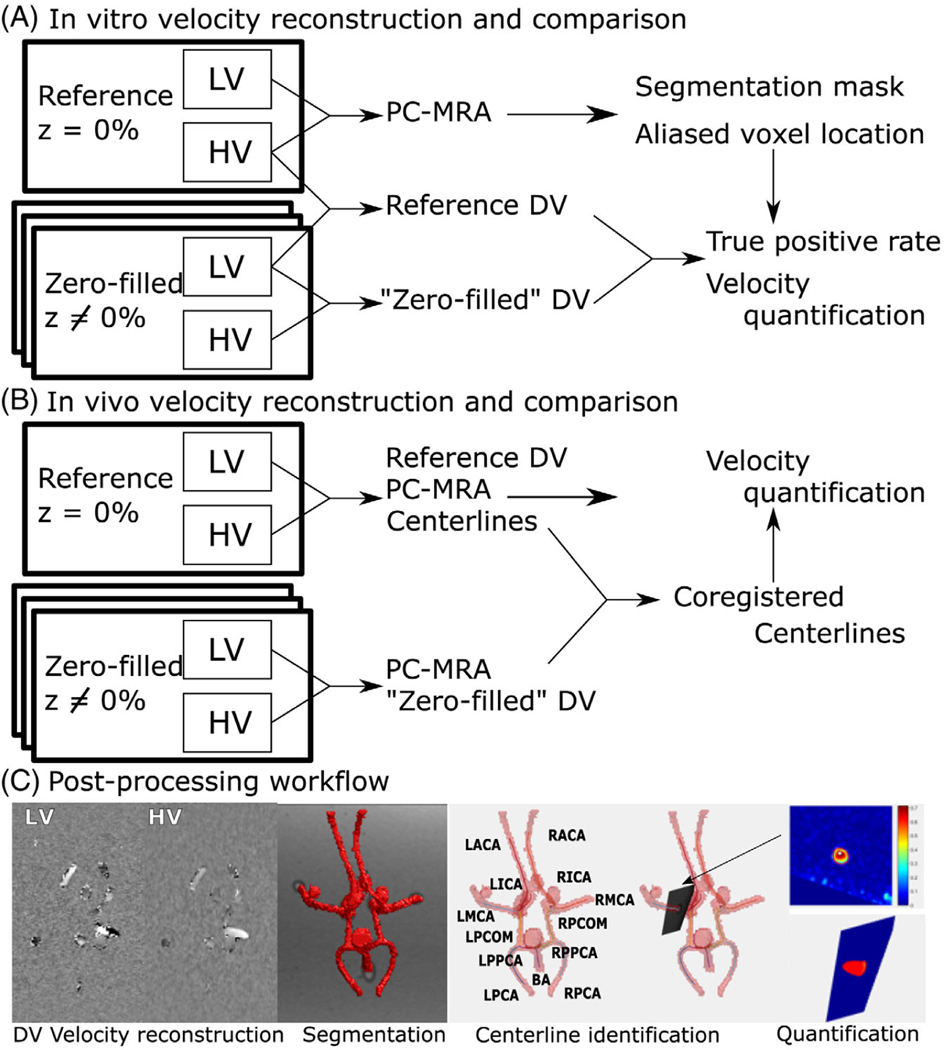
A, In vitro DV reconstruction and data comparison schematic for antialiasing performance and hemodynamic quantification performance. B, In vivo DV reconstruction and volumetric registration process schematic. C, Post-processing workflow for anatomical phantoms: velocity reconstruction, vessel segmentation, automated centerline extraction, and plane placement, plane-wise flow, and velocity quantification

For each value of *R*_Total_, two DV images, a “Zero-Filled DV” and a “Reference DV”, are reconstructed as in Figure 3A. The Zero-Filled DV is generated by correcting the LV from a given acquisition with the zero-filled HV data from the same acquisition (same $R_{\text{PEAK-GRAPPA}}$ and same value of *z*). The same LV data as in the Zero-Filled DV are separately corrected with the HV data from the reference acquisition (same $R_{\text{PEAK-GRAPPA}}$ and *z* = 0%) to generate a Reference DV dataset for each acquisition. Each pair of Zero-Filled and Reference DV datasets, therefore, has the same underlying velocity distribution of the LV, so any differences are due to varying effectiveness in antialiasing with HV. This is to enable voxel-by-voxel analysis of antialiasing effectiveness and quantification accuracy in vitro.

### 2.8 | In vivo image generation

For each of the VSRDV acquisitions with reduced spatial resolution HV, in vivo DV images were reconstructed from the LV and DV images from the acquisition with that *z* value as in Figure 3B.

### 2.9 | In vitro data analysis and statistics

Phase offset correction was performed by subtracting a first-order estimated background phase from uncorrected images, using a published method and an in-house tool.^34,35^ As in Figure 3C, a manually segmented angiogram mask based on the pseudo-complex difference phase-contrast MR angiogram^36^ (PCMRA) was generated from the reference dataset with $R_{\text{PEAK-GRAPPA}}=2$ and applied to all datasets. In order to avoid spurious results from comparing individual voxels at the boundaries of the PCMRA-based mask, the PCMRA mask was eroded by two voxels. The antialiasing effectiveness of the HV is assessed by the true positive rate (TPR), defined as the percentage of voxels within the eroded PCMRA mask that were categorized as aliased in the Reference DV and correctly identified as aliased in the Zero-Filled DV. To assess the effect of antialiasing differences on hemodynamic quantification, mean and peak velocity measurements across multiple planes in each imaging volume were computed using a previously published semi-automated workflow, including a secondary planewise segmentation^6^ with the original PCMRA mask used as input. Results of Bland–Altman analysis of plane-by-plane results are visualized as Flow Distribution Network Graphs (FDNGs),^22^ a schematic representation of the in vitro anatomy. Relationship between *z*, $R_{\text{PEAK-GRAPPA}}$ and cross-sectional area on differences between ZF and Reference in mean and peak velocity (Δmean and Δpeak, respectively) for each plane was analyzed with analysis of variance (ANOVA). Noise is characterized for LV, HV, and DV by the ratio of the average velocity value in static tissue and venc.

### 2.10 | In vivo data analysis and statistics

To account for subject displacement between acquisitions, a rigid registration of the PCMRA masks generated from the LV data of the Reference and Zero-Filled DV was performed as in Figure 3B using FSL FLIRT.^37,38^ The resulting transformation was applied to a set of centerlines extracted from the Reference DV, which was then used to calculate cross-sectional analysis planes, perform velocity quantification and extract a resulting FDNG as in Figure 3C. For comparison, the quantification workflow was also performed without registration. An experienced operator (M.A.) reviewed each plane with the image magnitude projected on it, to exclude from analysis any planes that intersected junctions between vessels or where the automated segmentation did not match the magnitude intensity.

Bland–Altman comparison of mean and peak velocity measurements across multiple planes in each imaging volume was performed in vivo. Results for agreement with *z* = 0% from the same session (for all 18 sessions with *z* = 40% and 80%) and test–retest reproducibility between sessions (for eight subjects with two sessions each) are visualized as group-level Flow Distribution Network Graphs for each value of *z* (all with $R_{\text{PEAK-GRAPPA}}=5$). Relationship between *z*, cross-sectional area and segmentation registration on Δmean and Δpeak for each vessel and subject was analyzed with ANOVA. Test–retest reproducibility is quantified using the repeatability coefficient.^39^

## 3 | RESULTS

### 3.1 | In vitro antialiasing performance

**FIGURE 4:**
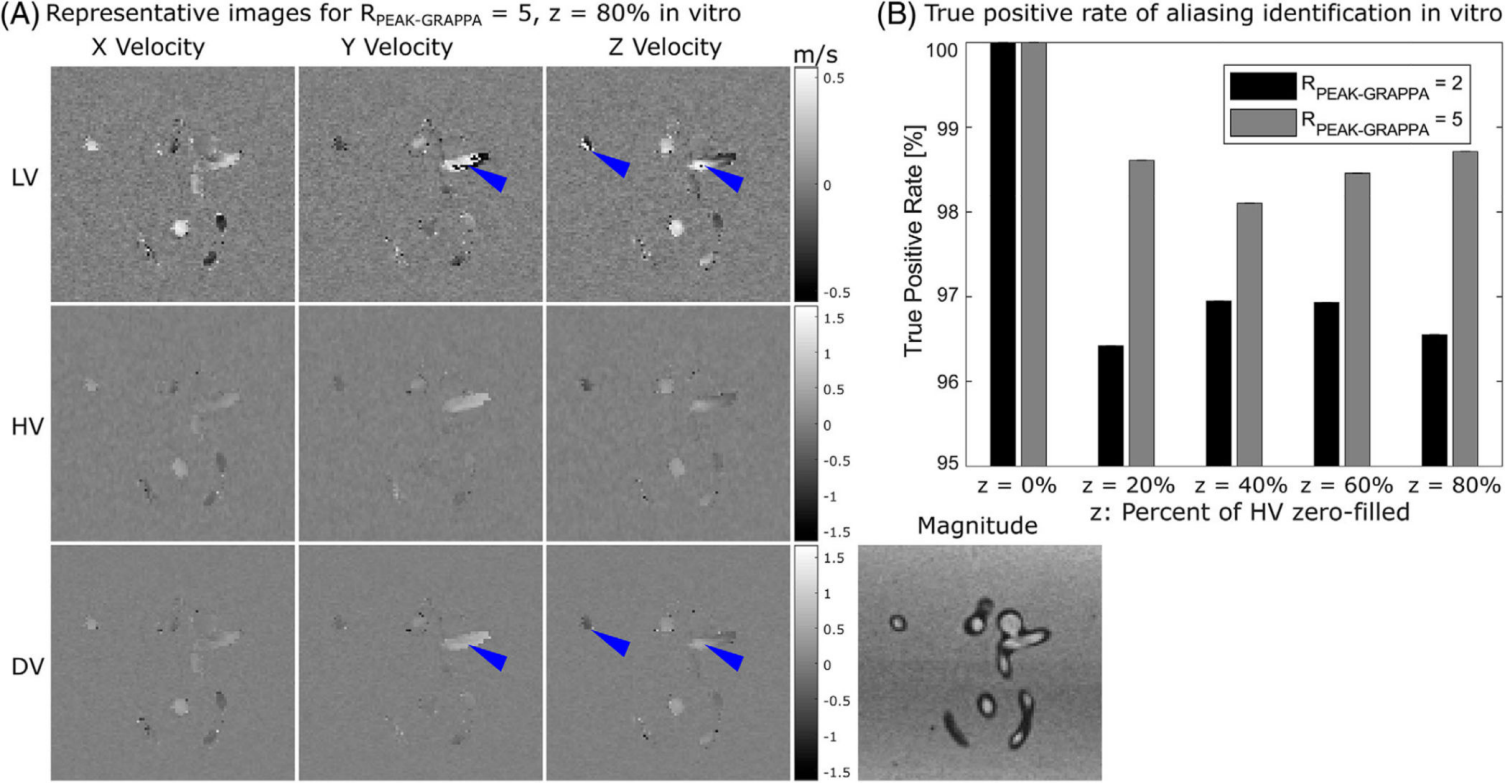
A, Representative images for in vitro data, including LV, HV, and Zero-filled DV for *R*_PEAK−GRAPPA_=5 and *z* = 80%. Blue markers indicate regions of aliasing in LV and their correction in the DV images. B, Antialiasing true positive rate

VSRDV images were obtained of the anatomical phantom at all combinations of *R*_PEAK−GRAPPA_ = 2 and 5 and *z* = 0%, 20%, 40%, 60%, and 80%, resulting in a maximum *R*_total_ of 5.7. In each case, no aliasing was visually observed in the flow regions for either HV or the Zero-Filled DV (Figure 4A).

The PCMRA mask contained 52896 velocity values, of which 625±38 were found to be aliased in the LV for scans with $R_{\text{PEAK-GRAPPA}}=2$ and 565±20 with $R_{\text{PEAK-GRAPPA}}=5$. For DV reconstructions at *z* = 0%, 571 voxels were identified as aliased in the LV for $R_{\text{PEAK-GRAPPA}}=2$ and 541 for $R_{\text{PEAK-GRAPPA}}=5$. When the HV from that acquisition was applied to the LV of other acquisitions to generate Reference DV images, the number of voxels identified as aliased increased by an average of 42±38 for $R_{\text{PEAK-GRAPPA}}=2$ and 21±20 for $R_{\text{PEAK-GRAPPA}}=5$. There was no relationship between *z* and the number of additional voxels identified as aliased (*p* = 0.87 for $R_{\text{PEAK-GRAPPA}}=2$ and 0.99 for $R_{\text{PEAK-GRAPPA}}=5$).

In identification of aliased voxels from the Reference DV in the Zero-Filled DV, the TPR ranged from 96.42% to 96.95% for $R_{\text{PEAK-GRAPPA}}=2$ and from 98.10% to 98.71% for $R_{\text{PEAK-GRAPPA}}=5$.^40^ There was no relationship between *z* and TPR (*p* = 0.21 and 0.28, respectively, for $R_{\text{PEAK-GRAPPA}}=2$ and 5).

### 3.2 | In vitro hemodynamic quantification

**FIGURE 5:**
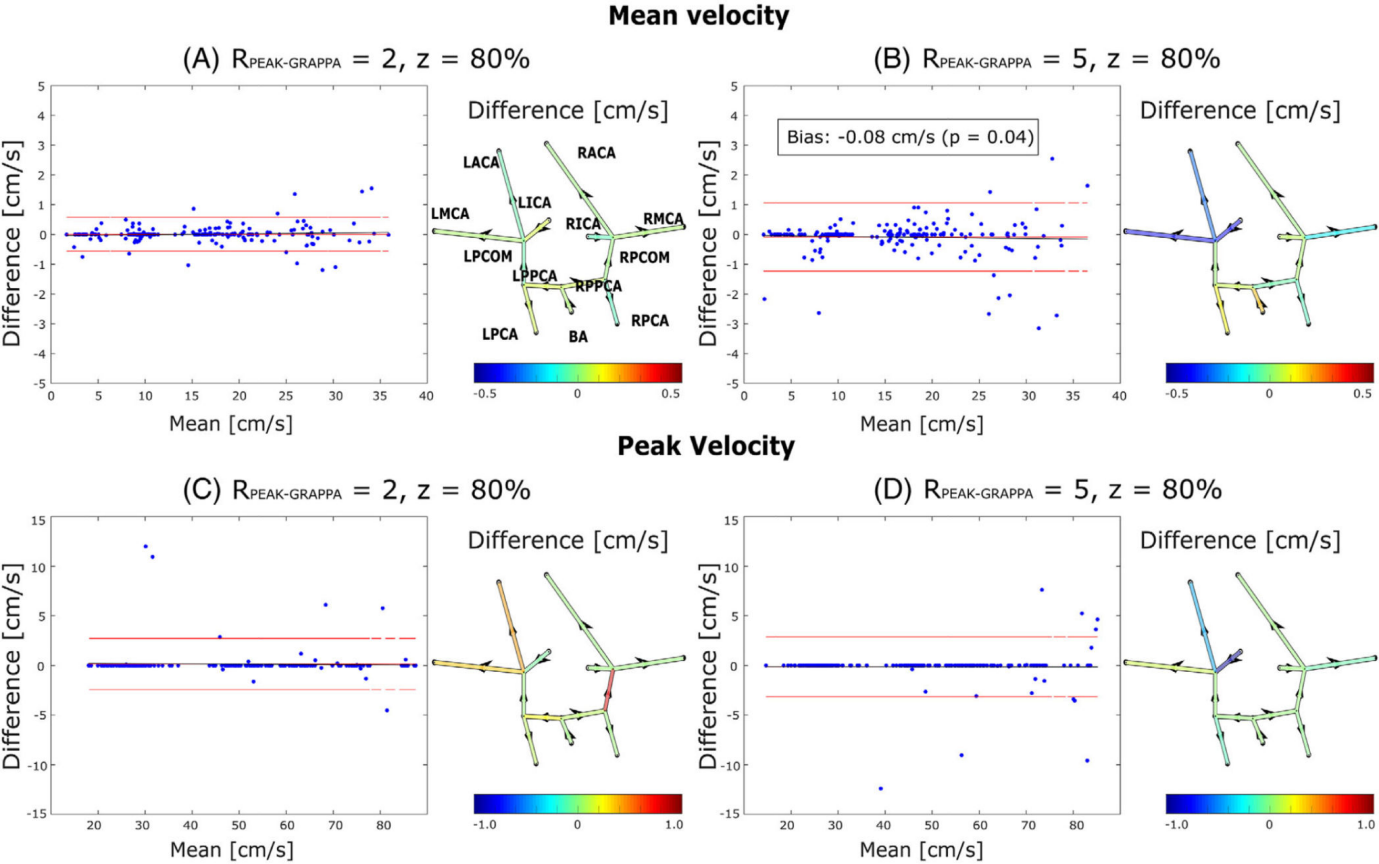
For mean velocity (cm/s) at *R*_PEAK−GRAPPA_ = 2 (A) and *R*_PEAK−GRAPPA_ = 5 (B), and for peak velocity (cm/s) at *R*_PEAK−GRAPPA_ = 2 (C) and *R*_PEAK−GRAPPA_ 5 (D), all with *z* = 80%, Bland–Altman plots for all cross-sectional plane locations in the entire anatomy, and in vitro FDNG showing the anatomical distribution of differences (Bland–Altman bias), between Reference and Zero-Filled DV. Bias and offset are indicated on the Bland–Altman plot where statistically significant. Vessels are labeled in (A)

Mean and peak velocities were computed based on each Zero-Filled DV and its corresponding Reference DV for all segmented vessels. Mean and peak velocities through time at a single plane are shown in Supporting Information Figure S1, which is available online. For all values of *R*_total_, the results of plane-wise Bland–Altman analysis for time-averaged values of each of the 13 vessels are summarized in the form of an FDNG, as shown in Figure 5A–D for the exemplary cases of *z* = 80% and $R_{\text{PEAK-GRAPPA}}=2$ and 5. Over all conditions tested, individual vessel mean velocity bias was 0.04±0.19cm/s and was highest (1.14cm/s) in the RMCA at $R_{\text{PEAK-GRAPPA}}=2$ and *z* = 80%. Peak velocity bias was 0.09±0.68 cm/s and was highest (3.32cm/s) in the RICA at $R_{\text{PEAK-GRAPPA}}=2$ and *z* = 40%. None of the in vitro model’s 13 vessels had statistically significant bias or offset in the Zero Filled DV relative to Reference DV in mean or peak velocity at any *R*_*ZF*_ value. Across all 13 vessels, Bland–Altman analysis was conducted as shown in Figure 5 for the exemplary cases of *z* = 80% and $R_{\text{PEAK-GRAPPA}}=2$ and 5. Results for all conditions tested are tabulated in Table 2. ANOVA analysis shows no statistically significant relationship between *R*, *z*, or cross-sectional area and Δpeak. There is a significant relationship between *z* (*p* = 0.01) and cross-sectional area (6×10^−4^) and Δmean; linear modeling shows a significant difference between *z* = 20% and *z* = 80% (*p* = 0.002) but no other *z* values. The magnitude of the change in Δmean is 0.017cm/s (0.02% of HV) per mm^2^.

### 3.3 | In vitro noise characteristics

As shown in Supporting Information Figure S2, LV velocity noise was 10.9% of venc at $R_{\text{PEAK-GRAPPA}}=2$ and 11.8% at $R_{\text{PEAK-GRAPPA}}=5$. DV velocity noise was 5.5% and 6.0% of venc at $R_{\text{PEAK-GRAPPA}}=2$ and 5, respectively, without significant variation with *z*.

### 3.4 | In vivo hemodynamic quantification

**FIGURE 6:**
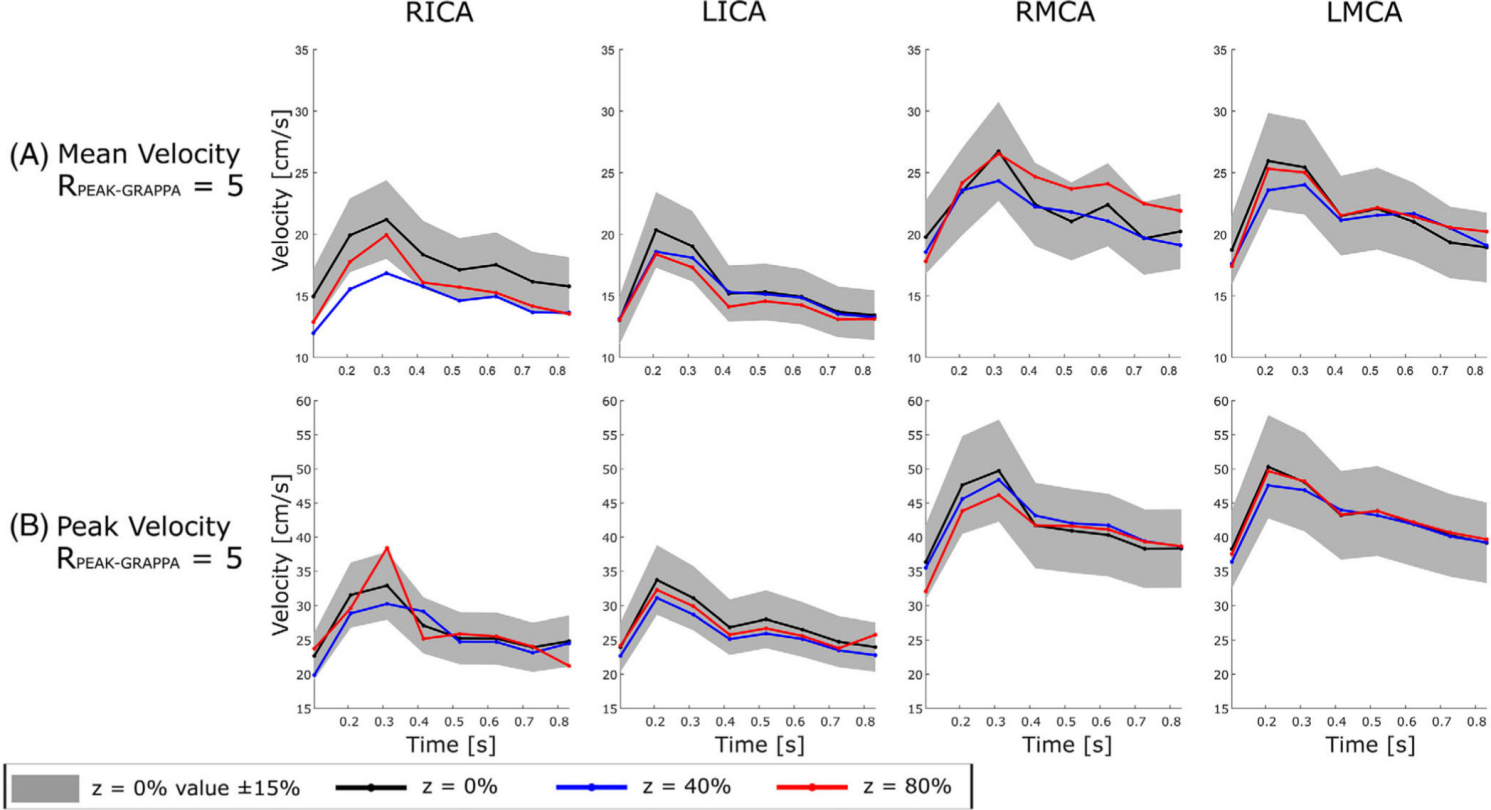
Time-resolved values at multiple individual planes are demonstrated for in vivo measurements of mean velocity (A), and (B) peak velocity, both at *R*_PEAK−GRAPPA_ = 5. Value of *z* is indicated by line color. Vessels are: RICA, right internal carotid artery; LICA, left internal carotid artery; RMCA, right middle cerebral artery; LMCA, right middle cerebral artery

**FIGURE 7:**
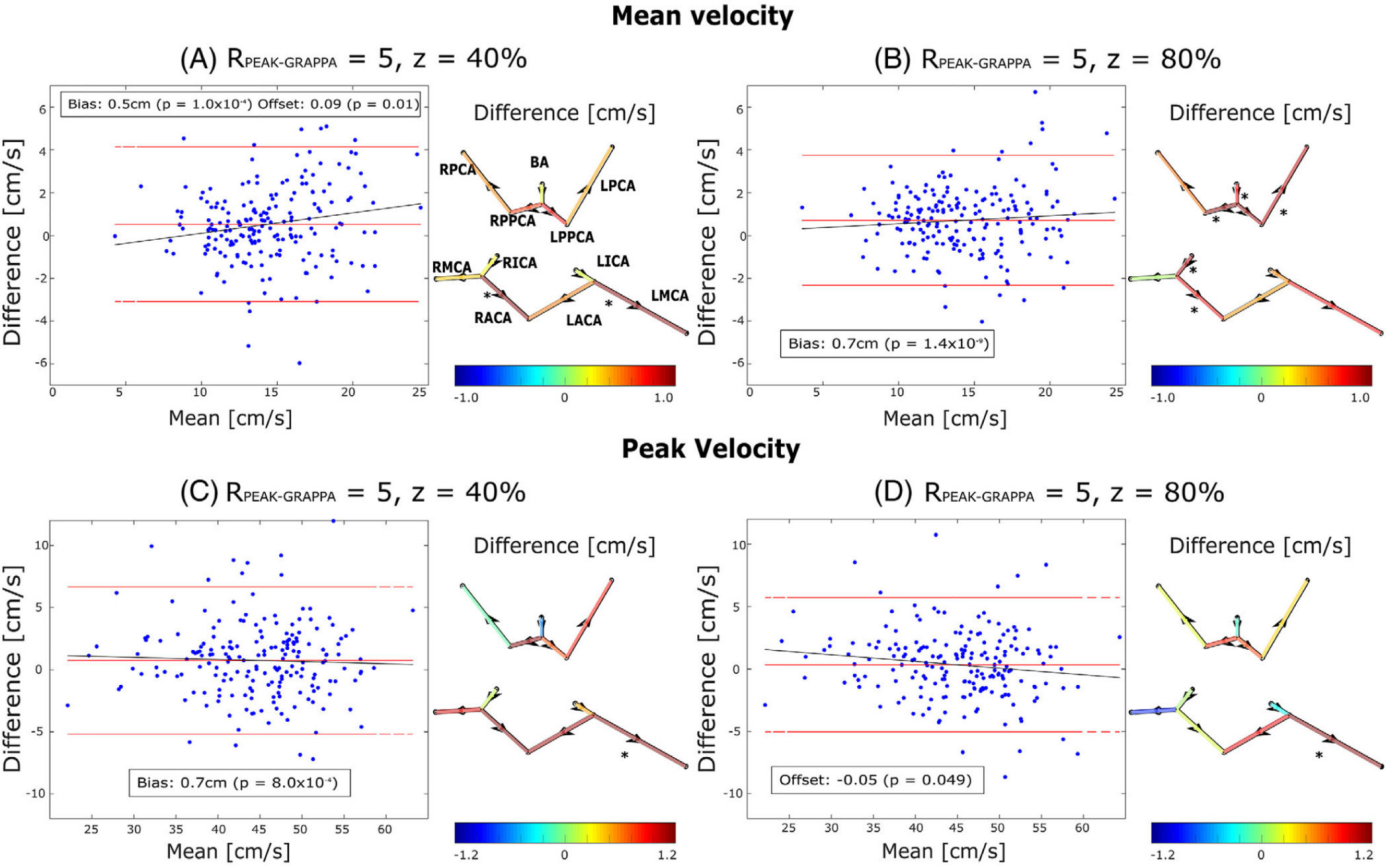
For mean velocity (cm/s) at *R*_PEAK−GRAPPA_ = 5, *z* = 40% (A) and *R*_PEAK−GRAPPA_ = 5, *z* = 80% (B), and for peak velocity (cm/s) at *R*_PEAK−GRAPPA_ = 5 *z* = 40% *R*_PEAK−GRAPPA_ = 5, *z* = 80% (D). Bland–Altman plots for all cross-sectional plane locations in the entire anatomy, and in vivo FDNG showing the anatomical distribution of differences (Bland–Altman bias), relative to *R*_PEAK−GRAPPA_ = 5, *z* = 0%. Bias and offset are indicated on the Bland–Altman plot where statistically significant. Vessels are labeled in (A)

Images with *z* = 0%, 40% and 80% and $R_{\text{PEAK-GRAPPA}}=5$ were acquired at all scan sessions. Mean and peak velocities were computed for all segmented vessels based on the DV at each *z*. Values through time at a single plane are shown in Figure 6. For all values of *R*_total_ the results of plane-wise Bland–Altman analysis for time-averaged values of mean and peak velocity compared to DV with *z*= 0% are summarized in the form of an FDNG, as shown in Figure 7. Of the 11 vessels identified in all subjects (not all subjects had RPCOM or LPCOM), bias relative to *z* = 0% was statistically significant for 2 vessels at *z* = 40% and 5 at *z*= 80% in mean velocity, and for 1 vessel at each of *z*= 40% and 80% for peak velocity. Over all conditions tested, the individual vessel mean velocity bias was 0.61±0.31cm/s and was highest (1.14cm/s) in the RPPCA at *z* = 20%. Peak velocity bias was 0.54±0.74 cm/s and was highest (2.1 cm/s) in the LMCA at *z* = 40%.

Across all 11 vessels, Bland–Altman analysis demonstrated limits of agreement of 49% and 41% for mean velocity and 26% and 24% for peak velocity at *z* = 40% and 80%, respectively (Figure 7). This equates to 7.22 and 6.06cm/s for mean velocity and 11.81 and 10.74 cm/s for peak velocity at *z* = 40% and 80%, respectively (Table 3A,B).

Bland–Altman analysis of area demonstrated limits of agreement of 56% and 55% at *z* = 40% and 80%, respectively, which equates to 6.57 and 6.56mm^2^ (Supporting Information Figure S3). ANOVA demonstrates no significant relationship between Δpeak and average cross-sectional area of the vessel (*p* = 0.78), or *z* (*p* = 0.15) and likewise for Δmean (*p* = 0.35 for cross-sectional area and *p* = 0.28 for *z*).

### 3.5 | In vivo test–retest analysis

Within the test–retest cohort, there was no significant change between sessions in either mean systolic and diastolic blood pressure (*p* = 0.11 and 0.60, respectively) or RR interval (*p* = 0.73). Across all 11 vessels, Bland–Altman analysis between the two scan sessions was conducted as in Supporting Information Figure S4 for *z* = 0%, 40% and 80%. Reproducibility coefficients, expressed as a percentage of the mean value at the first scan, were 75%, 90%, and 84%, respectively, for mean velocity, and 44%, 40%, and 48%, respectively, for peak velocity. These coefficients equate to 10.37, 12.25, and 10.99 cm/s, respectively, for mean velocity, and 19.38, 17.27, and 20.86 cm/s, respectively, for peak velocity (Table 3C). There is no Significant relationship between registration approach and Δpeak (*p*= 0.38) or Δmean (*p*= 0.13). Test–retest RPC was higher with registration than without (by at most 11.08% of mean value for peak velocity and at most 10.38% for mean velocity).

## 4 | DISCUSSION

### 4.1 | In vitro: antialiasing performance and quantification

VSRDV was successfully implemented, allowing up to 35.4% reduction of scan time at a given temporal resolution for DV 4D Flow MRI. The TPR of antialiasing was greater than 95% for all values of *z* tested at both $R_{\text{PEAK-GRAPPA}}=2$ and 5, and had no linear relationship to *z*. Timecourses of individual planes demonstrated close agreement in velocity quantification, although overestimation of velocity was noted in some timepoints for Zero-Filled DV. Bland–Altman analysis of mean and peak velocity values in individual vessels demonstrated that none of the in vitro model’s 13 vessels had a statistically significant bias in the Zero-Filled DV relative to Reference DV in either measurement at any *R*_ZF_ value tested in vitro, and that the limits of agreement were generally within 15% of the reference value, although some individual vessels exhibited a marked difference from the Reference DV. As illustrated by FDNGs, mean velocity errors are higher in smaller vessels, which is intuitive as the area directly impacts the mean velocity calculation. Meanwhile, the highest observed error in peak velocity is noted in the RICA. This is one of the widest but most tortuous vessels in the phantom, and likely contains complex flow patterns.

### 4.2 | In vivo: quantification and test–retest reproducibility

Timecourses of individual planes demonstrated close agreement in velocity quantification, with some timepoints showing under- or over-estimation of velocity by Zero-Filled DV, as in the RICA (with underestimation being consistent with effects of uncorrected aliasing). VSRDV with *z* = 40% and 80% resulted in peak velocity values within 15% of the mean value at *z* = 0%, and mean velocity values within 30%. Higher relative limits of agreement in the mean velocity are likely due in part to variability in segmentation: cross-sectional area could only be computed within about 30% for *z* = 40% or 80%. These ranges represent a difference of about 6 mm^2^; i.e., a matter of six to seven voxels. Because mean velocity is directly related to blood flow rate, blood flow measurements based on these data would be similarly affected. Still, limits of agreement were comparable to the retrospectively undersampled approach using PCVIPR (on the order of 17cm/s for peak velocity in cardiac great vessels).^26^

Test–retest reproducibility studies suggested repeated measures of mean and peak velocity at *z* = 0% were likely to fall within 37% and 22% of the true value, respectively. This illustrates that agreement between *z* = 40% or 80% and *z* = 0% within the same sessionis comparable to agreement between repeated scans with *z* = 0% for both mean and peak velocity. Moreover, test–retest reproducibility did not show an apparent trend relative to *z*, suggesting that variability between scans is not necessarily attributable to the zero-filling approach. Additionally, the registration process that enabled a more direct comparison within scan sessions may contribute to increased variability between sessions.

### 4.3 | Limitations and future work

This work has several important limitations. First, in an effort to narrow the parameter space to clinically relevant options, a single combination of venc values was considered with a ratio of venc_high_ = 110 cm/s to venc_low_ = 55 cm/s equal to 2:1. By increasing the ratio of venc_high_: venc_low_, in conjunction with an adjusted phase unwrapping scheme, it could be possible to further reduce noise or increase velocity dynamic range, but this possibility was not directly investigated here. This resulted in a low overall proportion of voxel values being aliased. The outlook for this work includes a systematic analysis of various ratio values. Antialiasing effectiveness in combination with other methods was also not assessed. Prior work comparing various approaches to reducing aliasing includes the comparison of dual-venc to HV,^17^ Laplacian to HV,^41^ divergence-freetoLaplacian,^42^ and a mutual comparison^43^ between standard dual-venc, optimized dual-venc^44^ and triple-venc.^45^ Future work could explore the additional value of using an algorithmic,^45–47^ optimization-based,^41,48^ or machine-learning-based^49,50^ approach in combination with dual-venc.

Additionally, the Reference DV computed for comparison in this work is an imperfect ground truth estimate, which results in a paradoxical improved performance at $R_{\text{PEAK-GRAPPA}}=5$ over $R_{\text{PEAK-GRAPPA}}=2$ in vitro. There are fewer total aliased voxels in the LV at $R_{\text{PEAK-GRAPPA}}=5$ than $R_{\text{PEAK-GRAPPA}}=2$, which is consistent with increased smoothing of velocities close to venc_low_. Meanwhile, the inherent sampling of k-space at $R_{\text{PEAK-GRAPPA}}=5$ is less dense than at $R_{\text{PEAK-GRAPPA}}=2$, so as a result of zero-filling the outer edges of k-space the total number of $k_{y}-k_{z}$ lines omitted is higher at $R_{\text{PEAK-GRAPPA}}=2$ than at $R_{\text{PEAK-GRAPPA}}=5$. HV datasets collected with and without zero-filling are more different from each other for $R_{\text{PEAK-GRAPPA}}=2$ than for $R_{\text{PEAK-GRAPPA}}=5$ as a result.

Some voxels were misclassified as aliased in the Reference DV, which occurs more often at $R_{\text{PEAK-GRAPPA}}=2$. This results in an underestimate of the TPR relative to the actual aliasing present in the LV data of the scan, so the performance identified here is a conservative estimate. Thus, when HV zero-filling is applied, the total number of identified voxels in the Zero-Filled DV is lower, but the percentage of Reference DV is higher, for $R_{\text{PEAK-GRAPPA}}=5$ than $R_{\text{PEAK-GRAPPA}}=2$. The ideal experiment to characterize contributions to data quality from PEAK-GRAPPA factor would be a comparison between $R_{\text{PEAK-GRAPPA}}=5$ and $R_{\text{PEAK-GRAPPA}}=2$, performed by undersampling the same exact data. However, as the k-space sampling pattern with $R_{\text{PEAK-GRAPPA}}=5$ cannot be obtained by retrospectively undersampling the pattern of $R_{\text{PEAK-GRAPPA}}=2$, a fully sampled dataset would have to be acquired to obtain both patterns.

In vivo data analysis was limited by the need to segment flow regions independently for each acquisition due to subject position changes. This includes the identification of the cross-sectional area, which contributes to greater uncertainty in mean velocity measurement compared to peak velocity which is relatively robust to area. One way to address this in future applications of the VSRDV sequence is to use the acquisition time savings to increase LV spatial resolution. Additionally, typical data processing with this workflow includes selection of cross-sectional analysis planes with parabolic flow profiles based on an inline visualization. In order to avoid bias in this study, plane selection for in vivo data in this experiment was done based on image magnitude, so planes may have been included in analysis for all datasets that would otherwise have been excluded in typical processing.

Finally, the sequence demonstrated here is a prototype demonstrating the variable-resolution dual-venc 4D flow MRI concept, and is subject to some limitations. This sequence is based on an eight-point encoding scheme, in order to enable straightforward calculation of phase differences for both LV and HV measurements. Seven-point encoding schemes (where the phase reference acquisition is shared between the LV and HV) are more efficient and result in a higher temporal resolution overall,^17^ so integration of 7TR approach with the HV undersampling approach shown here could enable additional flexibility intemporal resolution at the cost of non-uniform temporal resolution across k-space. Prior work has identified that, for neurovascular dual-venc 4D Flow MRI, temporal resolution may be reduced while maintaining good agreement in flow (although not peak velocity) values.^51^ This suggests that the undersampling burden could be distributed in both temporal and spatial dimensions in subsequent approaches. Given recent advances in radial and spiral4Dflowimaging^52,53–55^ and compressed sensing,^56–58^ it could be possible to further develop this approach through implementation of the VSRDV concept within a non-Cartesian framework. This would enable greater flexibility of both temporal and spatial resolution, with fewer constraints on the specific matrix size, and with potentially a more straightforward adaptation of the reconstruction process. Future developments can improve on the current sequence by implementing VSRDV within a seven-point encoding scheme, or exploring a view-sharing approach at the periphery of k-space in HV scans, potentially enabling further undersampling in the temporal domain.

## 5 | CONCLUSIONS

In conclusion, the VSRDV sequence was successfully implemented, and in vitro results showed anti-aliasing error rates less than 1% and TPRs above 95% for all zero-filling percentages tested at $R_{\text{PEAK-GRAPPA}}=2$ or 5. FDNG analysis showed that no significant bias was introduced by antialiasing in any vessels in vitro. In vivo analysis at *z* = 40% and 80% demonstrated agreement within 15% for peak velocity compared to the reference, although increased variability was observed in mean velocity. The relative magnitude of quantification variability is comparable to prior work in cardiac imaging. Moreover, test–retest reproducibility coefficients were comparable to Bland–Altman limits of agreement between undersampled and *z* = 0% scans, and reproducibility coefficients were similar for *z* = 0%, 40%, and 80%. HV k-space could thus be zero-filled up to *z* = 80%, which represents up to 34.8% reduction of scan time, with acceptable impact on image quality and quantification accuracy.

VSRDV provides an opportunity to acquire dual-venc 4D Flow MRI data with maximal temporal resolution and data quality while reducing scan times. As part of a comprehensive imaging and post-processing pipeline, this may increase the feasibility of including intracranial 4D Flow imaging in the assessment of complex neurovascular disease.

## Supplementary Material

supinfo **Figure S1** Time-resolved values at multiple individual planes are demonstrated for in vitro measurements of (A) mean velocity at R_PEAK−GRAPPA_=2, (B) mean velocity at RPEAK−GRAPPA=5, (C) peak velocity at R_PEAK−GRAPPA_=2, and peak velocity at R_PEAK−GRAPPA_=5. Value of *z* is indicated by line color. Vessels are: RICA – right internal carotid artery; RACA – right anterior cerebral artery; RMCA – right middle cerebral artery
**Figure S2** For in vitro data, noise ratio (mean static tissue velocity divided by venc) is shown at each value of Z (A). Mean values and linear relationship to *z* is tabulated in (B). The noise ratio for HV acquisitions is listed only for *z* = 0 (that is, when HV resolution is equal to that of LV as in typical dual venc), as the noise ratio of the HV acquisition decreases with increasing values of *z*
**Figure S3** For area (mm^2^) at (A) R_PEAK−GRAPPA_=5, *z* = 40% (B) R_PEAK−GRAPPA_=5, *z* = 80%, Bland–Altman plots for all cross-sectional plane locations in the entire anatomy, and in vivo FDNG showing the anatomical distribution of differences (Bland–Altman bias), relative to R_PEAK−GRAPPA_=5, *z* = 0%. Bias and offset are indicated on the Bland–Altman plot where statistically significant. Vessels are labeled in (A).
**Figure S4** For mean velocity (cm/s) at (A) *z* = 0% (B) *z* = 40% (C) *z* = 80%, and for peak velocity (cm/s) at (D) *z* = 0% (E) *z* = 40% (F) *z* = 80%, all with R_PEAK−GRAPPA_=5, Bland–Altman plots for all cross-sectional plane locations in the entire anatomy for the second relative to first scan session. Bias and offset are indicated on the Bland–Altman plot where statistically significant

## Figures and Tables

**TABLE 1:** Scan parameters for in vitro acquisitions, with ACS_*y*_ = 40 and ACS_*z*_ = 8, matrix size 160×160×30, temporal resolution 104 ms

				Time savings versus *z* = 0%	
Reference acquisition	Experiment	Zero-filling fraction *n* (%)	*R*_total_	Time (min)	Percentage
*R*_PEAK-GRAPPA_ = 2, scan time 21.6 min	Reference: *z* = 0%	0	1.9	0	0
	Zero-Filled: *z* > 0%	20	2.1	2.1	9.8
		40	2.4	4.2	19.6
		60	2.6	6.2	28.6
		80	2.9	7.5	34.8
*R*_PEAK-GRAPPA_ = 5, scan time 10.1 min	Reference: *z* = 0%	0	4.1	0	0
	Zero-Filled: *z* > 0%	20	4.4	0.8	8.3
		40	4.9	1.7	16.6
		60	5.4	2.4	24.2
		80	5.7	3.0	29.5

*Note:* Scan time savings are calculated based on the 1020 ms pulse duration of the pump.

**TABLE 2:** Bland–Altman analysis results for mean and peak velocity, aggregated across all in vitro analysis planes

		Mean velocity					Peak velocity				
	*Z* (%)	Bias (cm/s)	Bias (% venc)	Bias *p*-value	Limits of agreement (% mean)		Bias (cm/s)	Bias (% venc)	Bias *p-*value	Limits of agreement (% mean)	
*R*_PEAK-GRAPPA_ =2	20	**0.12**	0.11	0.003	−6.8	8.4	0.07	0.063	0.521	−6.7	7.0
	40	0.06	0.055	0.108	−6.5	7.2	−0.07	−0.063	0.739	−13.3	13.0
	60	0.04	0.036	0.185	−5.1	5.6	−0.02	−0.018	0.906	−8.4	8.4
	80	0.01	0.0091	0.721	−3.7	3.8	0.15	0.013	0.093	−5.2	5.8
*R*_PEAK-GRAPPA_ =5	20	0.03	0.027	0.417	−7.3	7.7	0.08	0.073	0.765	−16.9	17.2
	40	0.04	0.036	0.349	−7.4	7.9	**0.34**	0.31	0.047	−10.1	11.7
	60	0.04	0.036	0.405	−7.9	8.4	0.04	0.036	0.857	−14.7	14.9
	80	**−0.08**	0.072	0.044	−8.0	6.9	−0.13	−0.12	0.230	−7.0	6.4

*Note:* Upper and lower limits of agreement are given as a percentage of the mean reference value.

**TABLE 3:** Bland–Altman analysis results for (A) mean and peak velocity and (B) cross-sectional area, aggregated across all in vivo analysis planes and all 18 scan sessions. (C) Test–retest repeatability coefficient (RPC, eight subjects) for mean and peak velocity. Statistically significant values are bold.

A														
	Mean velocity							Peak velocity						
z (%)	Bias (cm/s)	Bias (% vene)	Bias *p*-value	Offset (cm/s)	Offset *p* -value	LOA (%)		Bias (cm/s)	Bias (% vene)	Bias *p*-value	Offset (cm/s)	Offset *p*-value	LOA (%)	
40	**0.53**	**0.48**	1.00 × l0^−4^	0.09	0.01	−21.03	28.12	**0.74**	**0.67**	8.2 × 10^−4^	−0.02	0.58	−11.48	14.75
80	**0.71**	**0.65**	1.39 × l0^9^	0.04	0.24	−15.84	25.48	0.34	0.31	0.08	**−0.05**	0.05	−11.17	12.68
B														
	Cross-sectional area													
z (%)	Bias (mm^2^)		Bias (% vene)		Bias *p*-value			Offset (mm^2^)		Offset *p*-value		LOA (%)		
40	0.12		0.11		0.32			0.04		0.08		−26.99	29.02	
80	−0.50		−0.45		5.40 × 10^−5^			−0.01		0.57		−32.15	23.70	
C														
	Mean velocity						Peak velocity							
Z (%)	Bias (cm/s)	Bias (% vene)	Bias *p*-value	Offset (cm/s)	Offset *p*-value	RPC (%)	Bias (cm/s)		Bias (% vene)	Bias *p*-value	Offset (cm/s)	Offset *p*-value		RPC (%)
0	**−1.92**	**−1.75**	1.89 × l0^−9^	**−0.20**	0.02	75.25	**−1.35**		**−1.23**	0.01	−2.1 × 10^−3^	0.98		43.94
40	**−1.14**	**−1.04**	1.04 × 10^−3^	−0.02	0.88	90.17	0.53		0.48	0.27	0.04	0.53		39.59
80	**−1.83**	**−1.67**	3.44 × l0^−8^	−0.08	0.38	83.80	**−1.98**		**−1.80**	8.87 × l0^−4^	0.10	0.21		48.08

*Note:* Lower and upper 95% limits of agreement (LOA) are given as a percentage of the mean reference value.

## References

[1] Schnell S, Ansari SA, Vakil P, Characterization of cerebral aneurysms using 4D FLOW MRI. J Cardiovasc Magn Reson. 2012;14:W2.

[2] Brina O, Bouillot P, Reymond P, How flow reduction influences the intracranial aneurysm occlusion: a prospective 4D phase-contrast MRI study. Am J Neuroradiol. 2019;40:2117–2123. 31727755 10.3174/ajnr.A6312 PMC6975363

[3] Futami K, Uno T, Misaki K, Identification of vortex cores in cerebral aneurysms on 4D flow MRI. Am J Neuroradiol. 2019;40:2111–2116. 31753836 10.3174/ajnr.A6322 PMC6975340

[4] Orita E, Murai Y, Sekine T, Four-dimensional flow MRI analysis of cerebral blood flow before and after high-flow extracranial–intracranial bypass surgery with internal carotid artery ligation. Neurosurgery. 2018; 85:58–64. 10.1093/neuros/nyy192 29757425

[5] Hope TA, Purcell DD, von Morze C, Vigneron DB, Alley MT, Dillon WP. Evaluation of intracranial stenoses and aneurysms with accelerated 4D flow. Magnetic Resonance Imaging. 2010;28(1):41–46. 19577400 10.1016/j.mri.2009.05.042

[6] Vali A, Aristova M, Vakil P, Semi-automated analysis of 4D flow MRI to assess the hemodynamic impact of intracranial atherosclerotic disease. Magnetic Resonance Medicine. 2019;82(2):749–762. 10.1002/mrm.27747 PMC6510639 30924197

[7] Wu C, Schnell S, Vakil P, In vivo assessment of the impact of regional intracranial atherosclerotic lesions on brain arterial 3D hemodynamics. Am J Neuroradiol. 2017;38:515–522. 28057635 10.3174/ajnr.A5051 PMC7959995

[8] Li Y, Chen H, He L, Hemodynamic assessments of venous pulsatile tinnitus using 4D-flow MRI. Neurology. 2018;91:e586–e593. 29997192 10.1212/WNL.0000000000005948

[9] Chang W, Loecher MW, Wu Y, Hemodynamic Changes in Patients with Arteriovenous Malformations Assessed Using High-Resolution 3D Radial Phase-Contrast MR Angiography. American Journal of Neuroradiology. 2012;33(8):1565–1572. 22499844 10.3174/ajnr.A3010 PMC6278605

[10] Yamada S, Watanabe Y, Oshima M, Miyake H. Perioperative evaluation of blood volume flow in high-flow cerebral arteriovenous malformation using phase-contrast magnetic resonance angiography. Interdiscip Neurosurg. 2015;2:72–75.

[11] Li CQ, Hsiao A, Hattangadi-Gluth J, Handwerker J, Farid N. Early Hemodynamic Response Assessment of Stereotactic Radiosurgery for a Cerebral Arteriovenous Malformation Using 4D Flow MRI. American Journal of Neuroradiology. 2018;39(4):678–681. 29371257 10.3174/ajnr.A5535 PMC7410784

[12] Ansari SA, Schnell S, Carroll T, Intracranial 4D flow MRI: toward individualized assessment of arteriovenous malformation hemodynamics and treatment-induced changes. Am J Neuroradiol. 2013;34:1922–1928. 23639564 10.3174/ajnr.A3537 PMC7965432

[13] Rivera-Rivera LA, Johnson KM, Turski PA, Wieben O. Pressure mapping and hemodynamic assessment of intracranial Dural sinuses and Dural arteriovenous fistulas with 4D flow MRI. Am J Neuroradiol. 2018;39:485–487. 29269408 10.3174/ajnr.A5494 PMC6013315

[14] Schuchardt FF, Kaller CP, Strecker C, Hemodynamics of cerebral veins analyzed by 2d and 4d flow mri and ultrasound in healthy volunteers and patients with multiple sclerosis. Journal of Magnetic Resonance Imaging. 2020;51(1):205–217. 31102341 10.1002/jmri.26782

[15] Berman SE, Clark LR, Rivera-Rivera LA, Intracranial arterial 4d flow in individuals with mild cognitive impairment is associated with cognitive performance and amyloid positivity (1875–8908 [Electronic]). 10.3233/JAD-170402 PMC5617115 28826187

[16] Turski P, Scarano A, Hartman E, Neurovascular 4D flow MRI (phase contrast MRA): emerging clinical applications. Neurovascular Imaging. 2016;2.

[17] Schnell S, Ansari SA, Wu C, Accelerated dual-venc 4D flow MRI for neurovascular applications. J Magn Reson Imaging. 2017;46:102–114. 28152256 10.1002/jmri.25595 PMC5464980

[18] Lee AT, Pike GB Pelc NJ. Three-point phase-contrast velocity measurements with increased velocity-to-noise ratio (0740–3194 [Print]). 10.1002/mrm.1910330119 7891526

[19] Elbaz MS, Aristova M, Ma L, Towards cerebral aneurysm rupture risk prediction using quantitative analysis by 4D flow MRI: intra-aneurysmal vortical blood flow and association to wall shear stress. Stroke. 2019;50.

[20] Schnell SA, Aristova M, Potts MB, Advantages of Dual-Venc 4D Flow MRI in the Evaluation of Cerebral Aneurysms. ISMRM, Paris, France, 2018; 1221.

[21] Christopher Muskat Joseph, Sean Rothenberger, Ahmadreza Baghaie, Susanne Schnell, Michael Markl, Craig Goergen, Rayz Vitaliy Cerebral Aneurysm Hemodynamic Comparison between Computational Fluid Dynamics and Dual-Venc 4D Flow MRI. 8th World Congress of Biomechanics. Dublin, Ireland; 2018.

[22] Aristova M, Vali A, Ansari SA, Standardized evaluation of cerebral arteriovenous malformations using flow distribution network graphs and dual-venc 4D flow MRI. J Magn Reson Imaging. 2019;50:1718–1730. 31070849 10.1002/jmri.26784 PMC6842032

[23] Marlevi D, Schollenberger J, Aristova M, Noninvasive quantification of cerebrovascular pressure changes using 4D flow MRI. Magn Reson Med. 2021;86:3096–3110. 34431550 10.1002/mrm.28928 PMC11421438

[24] Jung B, Ullmann P, Honal M, Bauer S, Hennig J, Markl M. Parallel MRI with extended and averaged GRAPPA kernels (PEAK-GRAPPA): optimized spatiotemporal dynamic imaging. J Magn Reson Imaging. 2008;28:1226–1232. 18972331 10.1002/jmri.21561

[25] Johnson KM, Markl M. Improved SNR in phase contrast velocimetry with five-point balanced flow encoding. Magn Reson Med. 2010;63:349–355. 20099326 10.1002/mrm.22202 PMC3418793

[26] Nett EJ, Johnson KM, Frydrychowicz A, Four-dimensional phase contrast MRI with accelerated dual velocity encoding. J Magn Reson Imaging. 2012;35:1462–1471. 22282344 10.1002/jmri.23588 PMC3343178

[27] Aristova M, Pang J, Ma L, Markl M, Schnell S. Dual-Venc Phase Contrast MRI with Increased Flow Encoding Efficiency. 28th ISMRM. Montréal, QC, Canada; 2019; 2420.

[28] Aristova M, Pang J, Ma Y, Markl M, Schnell S. Dual-Venc 4D Flow MRI: Balancing Image Quality and Scan Time: Balancing Image Quality and Scan Time. 29th ISMRM. Virtual; 2020; 3797.

[29] Bernstein MA, Shimakawa A, Pelc NJ. Minimizing TE in moment-nulled or flow-encoded two-and three-dimensional gradient-echo imaging. J Magn Reson Imaging. 1992;2:583–588. 1392252 10.1002/jmri.1880020517

[30] Schnell S, Markl M, Entezari P, k–t GRAPPA accelerated four-dimensional flow MRI in the aorta: effect on scan time, image quality, and quantification of flow and wall shear stress. Magn Reson Med. 2013;72:522–533. 24006309 10.1002/mrm.24925 PMC4414256

[31] Aristova M.4D flow MRI for the characterization for intracranial vascular networks. Biomed Eng (NY). Volume Ph.D. Evanston, Illinois, USA: Northwestern University; 2020. p. 114.

[32] Bernstein MA, Fain SB, Riederer SJ. Effect of windowing and zero-filled reconstruction of MRI data on spatial resolution and acquisition strategy. Journal of Magnetic Resonance Imaging. 2001;14(3):270–280. 11536404 10.1002/jmri.1183

[33] Rohrer M, Bauer H, Mintorovitch J, Requardt M, Weinmann HJ. Comparison of Magnetic Properties of MRI Contrast Media Solutions at Different Magnetic Field Strengths. Investigative Radiology. 2005;40(11):715–724. 16230904 10.1097/01.rli.0000184756.66360.d3

[34] Walker PG, Cranney GB, Scheidegger MB, Waseleski G, Pohost GM, Yoganathan AP. Semiautomated method for noise reduction and background phase error correction in MR phase velocity data. J Magn Reson Imaging. 1993;3:521–530. 8324312 10.1002/jmri.1880030315

[35] Bock J, Kreher BW, Hennig J, Markl M. Optimized pre-processing of time-resolved 2 D and 3 D phase contrast MRI data. In: Proceedings of the 15th Annual Meeting of ISMRM, Berlin, 2007 (abstract 3138); 2007.

[36] Bock J.Optimal Processing to Derive Static PC-MRA from Time-Resolved 3D PC-MRI Data. Vol 16. International Society for Magnetic Resonance in Medicine; 2008:3053.

[37] Jenkinson M, Bannister P, Brady M, Smith S. Improved optimization for the robust and accurate linear registration and motion correction of brain images. Neuroimage. 2002;17(2):825–41. 12377157 10.1016/s1053-8119(02)91132-8

[38] Jenkinson M, Smith S. A global optimisation method for robust affine registration of brain images (1361–8415 [Print]). 10.1016/s1361-8415(01)00036-6 11516708

[39] Vaz S, Falkmer T, Passmore AE, Parsons R, Andreou P. The case for using the repeatability coefficient when calculating test–retest reliability. PLoS One. 2013;8:e73990. 10.1371/journal.pone.0073990 PMC3767825 24040139

[40] Callaghan M.barwitherr(errors,varargin). MATLAB Central File Exchange; 2020. https://www.mathworks.com/matlabcentral/fileexchange/30639-barwitherr-errors-varargin.

[41] Loecher M, Schrauben E, Johnson KM, Wieben O. Phase unwrapping in 4D MR flow with a 4D single-step laplacian algorithm. J Magn Reson Imaging. 2016;43:833–842. 26417641 10.1002/jmri.25045

[42] Zhang J, Rothenberger SM, Brindise MC Divergence-Free Constrained Phase Unwrapping and Denoising for 4D Flow MRI Using Weighted Least-Squares. IEEE Transactions on Medical Imaging. 2021;40(12):3389–3399. 34086567 10.1109/TMI.2021.3086331 PMC8714458

[43] Franco P, Ma L, Schnell S, Markl M, Bertoglio C, Uribe S. A Review and Comparison of Unwrapping Methods in Dual Velocity-Encoding MRI. ISMRM. Virtual; 2022; 4400.

[44] Carrillo H, Osses A, Uribe S, Bertoglio C. Optimal Dual-VENC Unwrapping in Phase-Contrast MRI. IEEE Transactions on Medical Imaging. 2019;38(5):1263–1270. 30475716 10.1109/TMI.2018.2882553

[45] Ma LE, Markl M, Chow K, Vali A, Wu C, Schnell S. Efficient triple-VENC phase-contrast MRI for improved velocity dynamic range. Magnetic Resonance in Medicine. 2020;83(2):505–520. 31423646 10.1002/mrm.27943 PMC7051107

[46] Salfity MF, Huntley JM, Graves MJ, Marklund O, Cusack R, Beauregard DA. Extending the dynamic range of phase contrast magnetic resonance velocity imaging using advanced higher-dimensional phase unwrapping algorithms. J R Soc Interface. 2006;3:415–427. 16849270 10.1098/rsif.2005.0096 PMC1578755

[47] Wang K, Zhao S, Liu B, Perturbations of BMP/TGF-beta and VEGF/VEGFR signalling pathways in non-syndromic sporadic brain arteriovenous malformations (BAVM). 10.1136/jmedgenet-2017-105224. LID - (1468–6244 [Electronic]). PMC6161649 30120215

[48] Herment A, Mousseaux E, Jolivet O, Improved estimation of velocity and flow rate using regularized three-point phase-contrast velocimetry. Magn Reson Med. 2000; 44:122–128. 10893530 10.1002/1522-2594(200007)44:1<122::aid-mrm18>3.0.co;2-c

[49] Justice A.Phase Unwrapping of 4D-Flow MRI Data with Graph Cuts. Volume Master: University of Louisville; 2018.

[50] Fernandes JF, Faraci A, Rigolli M, Multiple phase unwrapping of 4D-flow MRI in cardiovascular valves and vessels. ISMRM. Virtual; 2020; 0663.

[51] Maria Aristova, Carr James, Ansari Sameer, Wu Can, Schnell Susanne. Fast and full coverage dual-venc 4D flow MRI: can time-averaged acquisition be useful? ISMRM Singapore; 2016.

[52] Ma LE, Yerly J, Piccini D, 5D Flow MRI: A Fully Self-gated, Free-running Framework for Cardiac and Respiratory Motion-resolved 3D Hemodynamics. Radiology: Cardiothoracic Imaging. 2020;2(6):e200219. 10.1148/ryct.2020200219 PMC7755133 33385164

[53] Morgan AG, Thrippleton MJ, Wardlaw JM, Marshall I. 4D flow MRI for non-invasive measurement of blood flow in the brain: a systematic review. J Cereb Blood Flow Metab. 2020; 41:206–218. 32936731 10.1177/0271678X20952014 PMC8369999

[54] Chang W, Landgraf B, Johnson KM, Velocity measurements in the middle cerebral arteries of healthy volunteers using 3D radial phase-contrast HYPRFlow: comparison with transcranial Doppler sonography and 2D phase-contrast MR imaging. AJNR Am J Neuroradiol. 2011;32:54–59. 20947642 10.3174/ajnr.A2240 PMC3133942

[55] Callahan S, Singam NS, Kendrick M, Dual-V enc acquisition for 4D flow MRI in aortic stenosis with spiral readouts. Journal of Magnetic Resonance Imaging. 2020;52(1):117–128. 31850597 10.1002/jmri.27004 PMC7299789

[56] Ma LE, Markl M, Chow K, Aortic 4D flow MRI in 2 minutes using compressed sensing, respiratory controlled adaptive k-space reordering, and inline reconstruction. Magnetic Resonance in Medicine. 2019;81(6):3675–3690. 30803006 10.1002/mrm.27684 PMC6535305

[57] Hinostroza V.4D Flow with Compressed Sensing for the Evaluation of Intracranial Aneurysmal Flow Patterns. Volume Master of Science: UCSF; 2019.

[58] Liu J, Koskas L, Faraji F, Highly accelerated intracranial 4D flow MRI: evaluation of healthy volunteers and patients with intracranial aneurysms. Magnetic Resonance Materials in Physics, Biology and Medicine. 2018;31(2):295–307. 10.1007/s10334-017-0646-8 PMC5803461 28785850

