# Supplementary material for: Accelerated dual-venc 4D flow MRI with variable high-venc spatial resolution for neurovascular applications
Source: Magn Reson Med. Author manuscript; Available in PMC 2023 Oct 1. (PMC9392495; doi:10.1002/mrm.29306)
Supplement: supinfo — Figure S1 Time-resolved values at multiple individual planes are demonstrated for in vitro measurements of (A) mean velocity at RPEAK−GRAPPA=2, (B) mean velocity at RPEAK−GRAPPA=5, (C) peak velocity at RPEAK−GRAPPA=2, and peak velocity at RPEAK−GRAPPA=5. Value of z is indicated by line color. Vessels are: RICA – right internal carotid artery; RACA – right anterior cerebral artery; RMCA – right middle cerebral artery Figure S2 For in vitro data, noise ratio (mean static tissue velocity divided by venc) is shown at each value of Z (A). Mean values and linear relationship to z is tabulated in (B). The noise ratio for HV acquisitions is listed only for z = 0 (that is, when HV resolution is equal to that of LV as in typical dual venc), as the noise ratio of the HV acquisition decreases with increasing values of z Figure S3 For area (mm2) at (A) RPEAK−GRAPPA=5, z = 40% (B) RPEAK−GRAPPA=5, z = 80%, Bland–Altman plots for all cross-sectional plane locations in the entire anatomy, and in vivo FDNG showing the anatomical distribution of differences (Bland–Altman bias), relative to RPEAK−GRAPPA=5, z = 0%. Bias and offset are indicated on the Bland–Altman plot where statistically significant. Vessels are labeled in (A). Figure S4 For mean velocity (cm/s) at (A) z = 0% (B) z = 40% (C) z = 80%, and for peak velocity (cm/s) at (D) z = 0% (E) z = 40% (F) z = 80%, all with RPEAK−GRAPPA=5, Bland–Altman plots for all cross-sectional plane locations in the entire anatomy for the second relative to first scan session. Bias and offset are indicated on the Bland–Altman plot where statistically significant [file NIHMS1803517-supplement-supinfo.pdf]

Supplementary figures

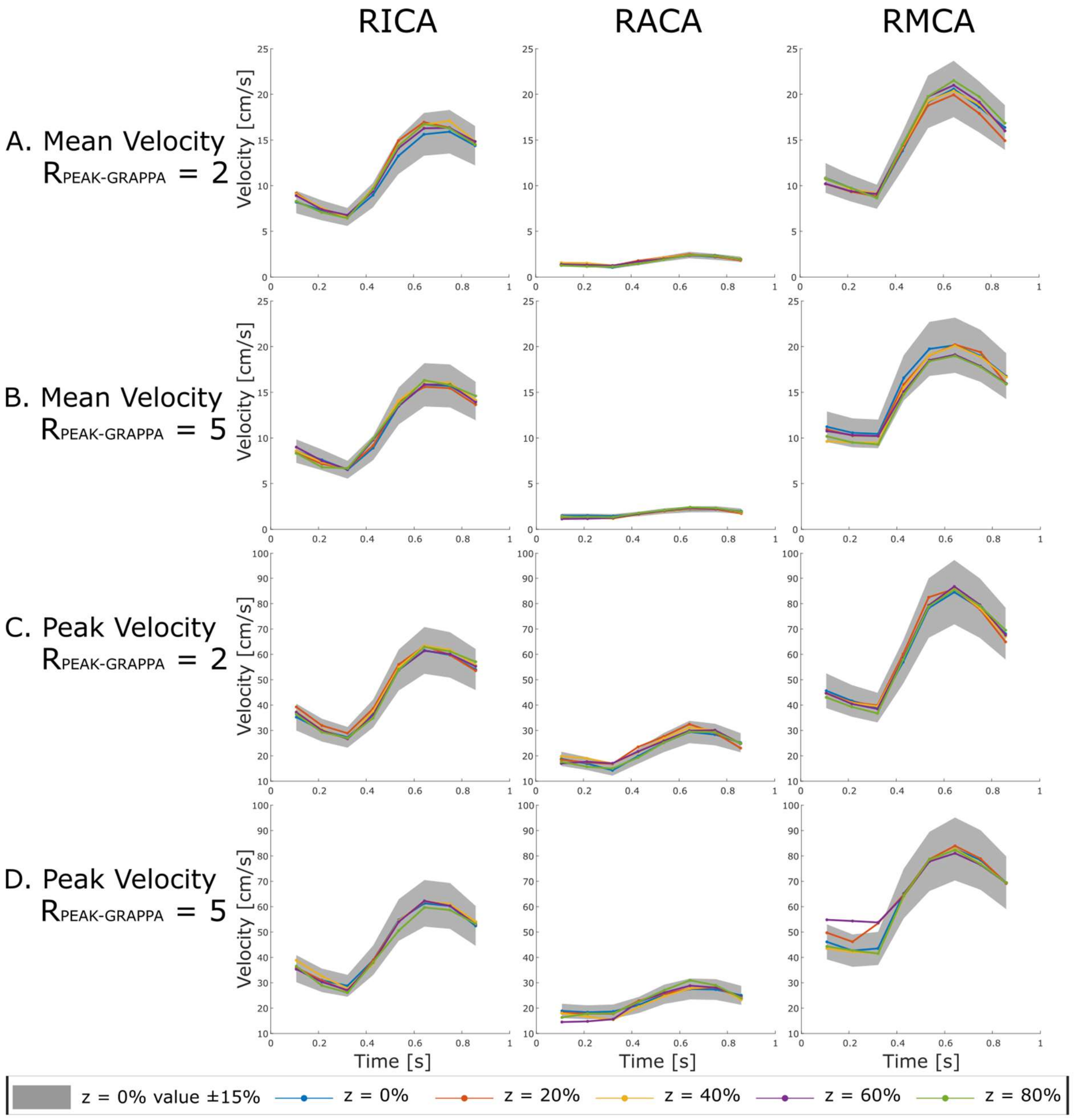

Figure S 1

Time-resolved values at multiple individual planes are demonstrated for in vitro measurements of (A) mean velocity at $R_{PEAK-GRAPPA}$= 2, (B) mean velocity at $R_{PEAK-GRAP}$ = 5, (C) peak velocity at $R_{PEAK-GRAPPA}$= 2, and peak velocity at $R_{PEAK-GRAPPA}$= 5. Value of z is indicated by line color. Vessels are: RICA – right internal carotid artery; RACA – right anterior cerebral artery; RMCA – right middle cerebral artery.

A. In vitro velocity noise in static tissue

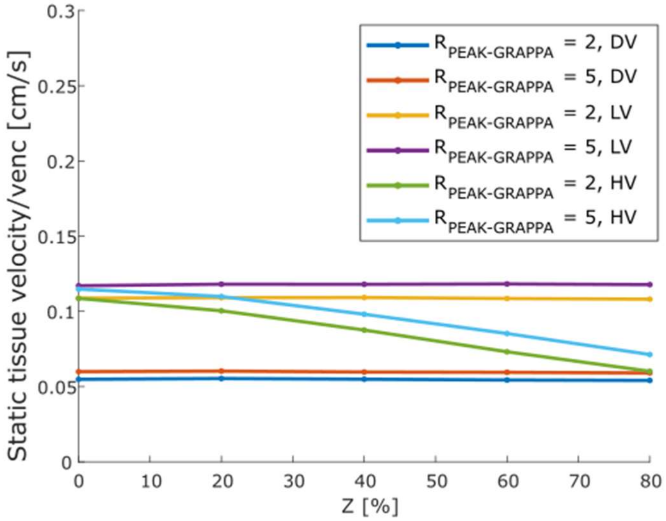

B. In vitro velocity noise linear fit vs z

Experiment	Noise ratio mean across Z values	P-value of relationship to Z
$R_{PEAK-GRAPPA}$ = 2, DV	5.5%	0.1070
$R_{PEAK-GRAPPA}$ = 5, DV	6.0%	0.0548
$R_{PEAK-GRAPPA}$ = 2, LV	10.9%	0.1882
$R_{PEAK-GRAPPA}$ = 5, LV	11.8%	0.3189
$R_{PEAK-GRAPPA}$ = 2, HV Z = 0% only	10.9%	--
$R_{PEAK-GRAPPA}$ = 5, HV Z = 0% only	11.5%	--

Figure S 2

For in vitro data, noise ratio (mean static tissue velocity divided by venc) is shown at each value of Z (A). Mean values and linear relationship to z is tabulated in (B). The noise ratio for HV acquisitions is listed only for z = 0 (that is, when HV resolution is equal to that of LV as in typical dual venc), as the noise ratio of the HV acquisition decreases with increasing values of z.

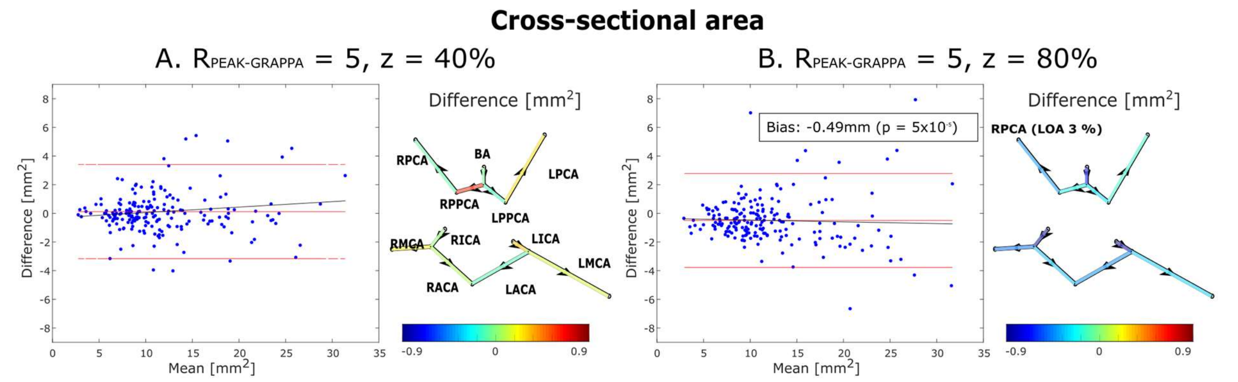

Figure S 3

For area (mm²) at (A) $R_{PEAK-GRAPP}$ *= 5, z = 40% (B)* $R_{PEAK-GRAPP}$ *= 5, z = 80%, Bland-Altman plots for all cross-sectional plane locations in the entire anatomy, and in vivo FDNG showing the anatomical distribution of differences (Bland-Altman bias), relative to* $R_{PEAK-GRAPPA}$*= 5, z = 0%. Bias and offset are indicated on the Bland-Altman plot where statistically significant. Vessels are labeled in (A).*

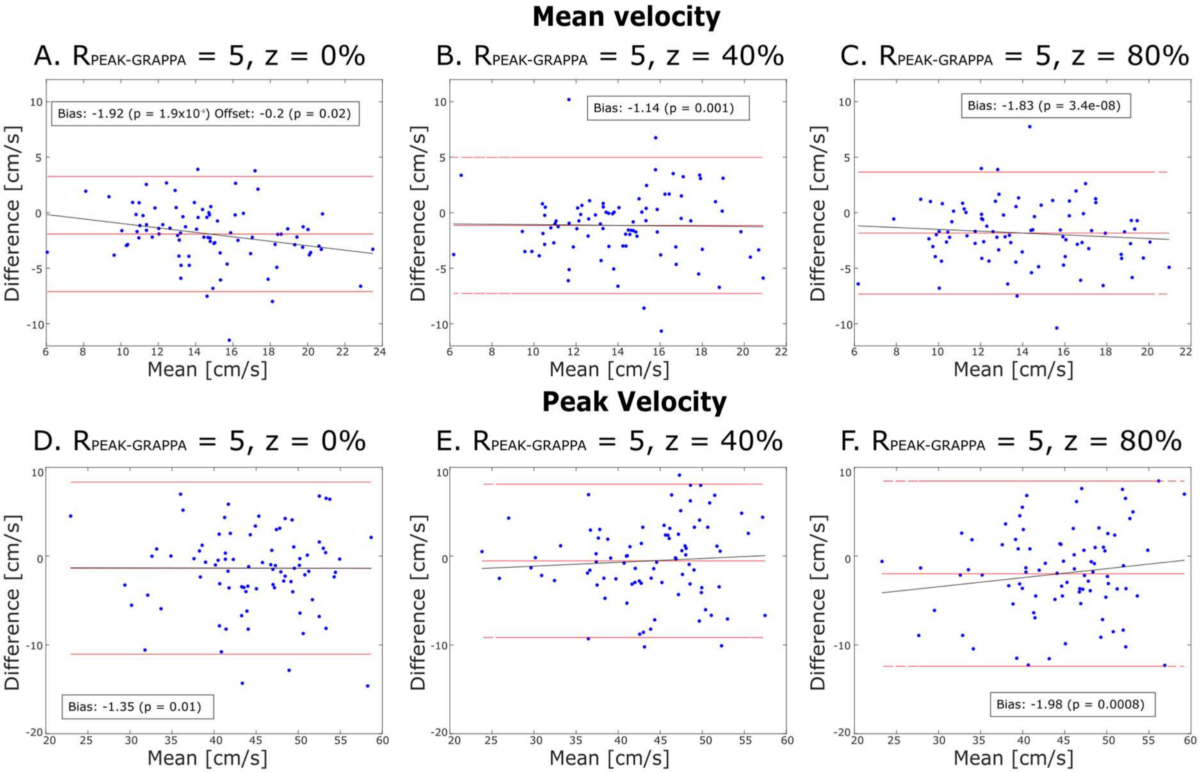

Figure S 4

For mean velocity (cm/s) at (A) z = 0% (B) z = 40% (C) z = 80%, and for peak velocity (cm/s) at (D) z = 0% (E) z = 40% (F) z = 80%, all with $R_{PEAK-GRAPPA}$= 5, Bland-Altman plots for all cross-sectional plane locations in the entire anatomy for the second relative to first scan session. Bias and offset are indicated on the Bland-Altman plot where statistically significant.
